# Ornament and Use

**Published:** 1875-02

**Authors:** 


### ORNAMENT AND USE.

There is a great furniture palace on West 23d street, near 6th avenue, in this city, kept by a couple of enterprising workers, the plan and method of which is worthy of notice. It may be told in a few words. It is simply to make every article sold sell some other article. This is accomplished by fair prices and perfect goods.

It is not difficult to cheat in furniture, but the deception is certain to make itself known. In our trying climate, with its great variations of temperature, the tests applied to furniture in use are very severe. If it is not well made, and of good material, the fact soon appears, and the reputation of the dealer suffers.

Messrs. Degraff & Cochrane, the manufacturers whom we have in mind, have hosts of silent salesmen constantly employed. Now it is a parlor set, elegant in design and strong in construction, the faithful service of which tells its own story of honest workmanship, and thus secures for the house new customers. Again it is a less pretentious outfit for the home of the mechanic, of whose strength and durability it is its own best witness.

New York boasts many great furniture houses, but we know of none which we would enter with greater certainty of finding just what we desired at prices which would leave us no single ground of complaint. It is not possible for us to assert of all our business houses, that customers at a distance will be dealt with as satisfactorily as will those who are on the spot; but in reference to this house we are able to make this assertion. Their stock comprises everything which can be thought of in furniture, house-decorations, bedding and upholstery, and it is a genuine pleasure to examine their substantial and elegant goods.

